# A Cu^2+^ triggered reversible photochromic system: the structure photochromic response relationship study and potential applications

**DOI:** 10.1098/rsos.230121

**Published:** 2023-06-07

**Authors:** Shuping Wang, Lumiao Wu, He Li, Guoyan Zhang, Xueqian Wei, Jiyi Zheng, Long Jiang, Ping Ju, Fengli Qu, Ensheng Zhang

**Affiliations:** ^1^ College of Chemistry and Chemical Engineering, Qufu Normal University, Qufu, Shandong 273165, People's Republic of China; ^2^ Instrumental Analysis & Research Center, Sun Yat-Sen University, Guangzhou 510275, People's Republic of China

**Keywords:** photochromic, Cu^2+^ trigger, structure photochromic response relationship, information storage

## Abstract

Fifteen rhodamine B hydrazide hydrazone (RhBHH) derivatives (compounds ***a***–***o***) with various substituent groups at different position and their photochromic property triggered by Cu^2+^ were studied to illustrate the structure photochromic response relationship (SPRR). Three of them (compounds ***f***–***h***) with a *para*-position hydroxyl group and two *meta*-position halogen substituents display Cu^2+^-triggered photochromic which is significantly different from the previous reports. It was found that halogen atoms, which were generally considered to have no remarkable regulation effect, exhibited great influences on the photochromic behaviour of RhBHH derivatives. Detail photochromic properties of the developed photochromic system were revealed by using compound ***g*** as the model substrate, and only Cu^2+^ displayed high selective trigger effect. Good reversible photochromic phenomenon was observed after stimulated with visible light irradiation and dark (or heat) bleaching consecutively. Furthermore, this photochromic system could be used in the preparation of photochromic glass, special security ink, molecular logic gate and two-dimensional code for security information storage.

## 1. Introduction

Photochromic material as a kind of stimuli-responsive material has drawn great attention in the past few years [1–4]. In most cases, the colour change of photochromic material was accomplished by reversible transformation of two isomers which have different absorption spectra [1]. The reversible transformation was started with light irradiation and reversed with thermal reversion (T-type photochromism) or another light irradiation (P-type photochromism) [3,5]. Due to their remarkable colour change after light stimulation, photochromic materials could be used in the preparation of molecular switches [2], optical data storage [6], molecular logic gates [6] and colorimetric chemo-sensors [7], etc. To date, organic photochromic materials with core structures such as diarylethene [8,9], rhodamine [10–13], spiropyran [14–16] and azobenzene [4,17] have been developed. Among those reported photochromic materials, rhodamine derivatives have received significant attention in consideration of their outstanding advantages such as simple synthesis, distinctive colour change and good recoverability.

Rhodamine B hydrazide hydrazone (RhBHH) as a typical xanthene derivate has been proved to be a wonderful scaffold for photochromic materials design in recent years [18–23]. Especially, the photochromic phenomenon of RhBHH derivatives could be triggered by different metal ions which made them more suitable for particular applications such as secure information storage. In 2014, Tang group [18] reported a rhodamine B salicylaldehyde hydrazine-based photochromic system which could be triggered by Ni^2+^, Zn^2+^ and Cd^2+^. In such photochromic system, the *ortho* hydroxyl group of salicylaldehyde played a key role to coordinate with metal ions. Therefore, salicylaldehyde or 2-pyridine carboxaldehyde derivatives which have an *ortho*-coordination site were employed for coupling with rhodamine B hydrazide to construct photochromic system in most of the follow-up studies [19–26]. Although great progress has been made in the development of photochromic materials [27–29], the structure photochromic response relationship (SPRR) of RhBHH-based photochromic materials are still blurry. In addition, due to the strong chelation of the above RhBHH derivatives, no photochromic phenomenon would be observed or too much bleaching time would be needed when Cu^2+^ was used as the trigger [18].

**Scheme 1.**
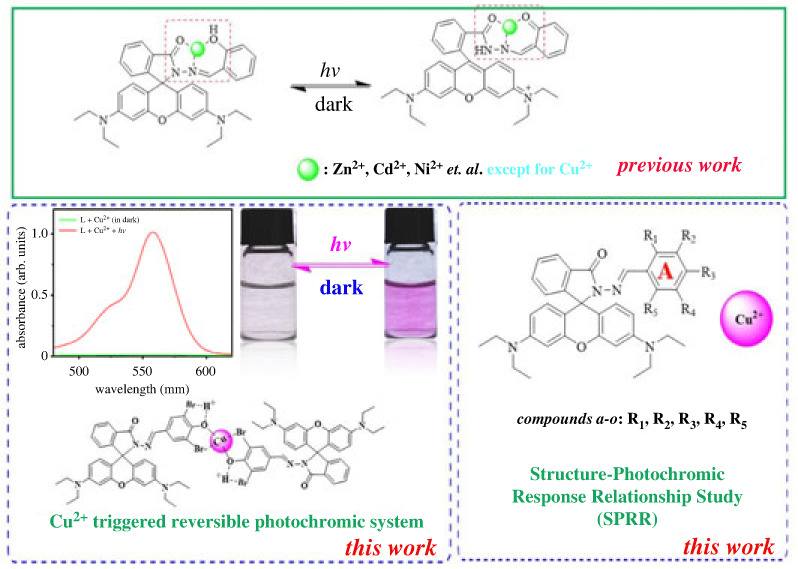
Previous works and our Cu^2+^ triggered reversible photochromic system as well as the SPRR study.

In this work, we intend to build a new photochromic system which could be triggered by Cu^2+^ and explore the SPRR of RhBHH-based photochromic materials. Fifteen RhBHH derivatives (electronic supplementary material, table S2 and table 1, compounds ***a***–***o***) with various substituent groups at different position of benzene ring (scheme 1, ring **A**) have been synthesized and their photochromic property triggered by Cu^2+^ was systematically studied. It was found that both the position and the kind of substituents (scheme 1 ring **A**, R_1_-R_5_; table 1, compounds ***a***–***o***) have great influence on the photochromic property of RhBHH derivatives. The main findings could be summarized as follows: ① reversible photochromic response of compounds ***f***–***h*** (R_1_, R_5_ = H, R_2_, R_4_ = X, R_3_ = OH, X = Cl, Br and I) could be selectively triggered by Cu^2+^ under visible light; ② Cu^2+^ is not the suitable trigger for RhBHH compounds that have a hydroxyl group at R_1_ position (***a***, R_1_, R_2_, R_3_ = OH, and ***o***, R_1_, R_3_ = OH) for irreversible colour change could be observed after treatment with Cu^2+^; ③ a hydroxyl group at R_3_ position and two halogen atoms at R_2_ and R_4_ position are the key structure for Cu^2+^ triggered photochromic RhBHH derivatives (R_2_, R_4_ = X, R_3_ = OH, X = Cl, Br and I). From the above results, we confirmed that new photochromic system triggered by Cu^2+^ could be obtained by changing the R_2_–R_4_ substituent groups (scheme 1, ring **A**), and halogen atoms displayed a key role in the modulation of photochromic property. In addition, the detail photochromic properties and potential application of the newly developed photochromic system was investigated by using compound ***g*** (R_2_, R_4_ = Br, R_3_ = OH) as the model substrate. The critical advantages of this system such as fast response, good fatigue resistance and moderated thermal bleaching rate made it a promising candidate material for information storage and encryption [30–32]. In addition, applications to use this photochromic system in the preparation of photochromic glass, special security ink, molecular logic gate and security information storage device were performed.

**Table 1.** General molecular structure of compounds ***a***–***o*** and a summary of their photochromic property.

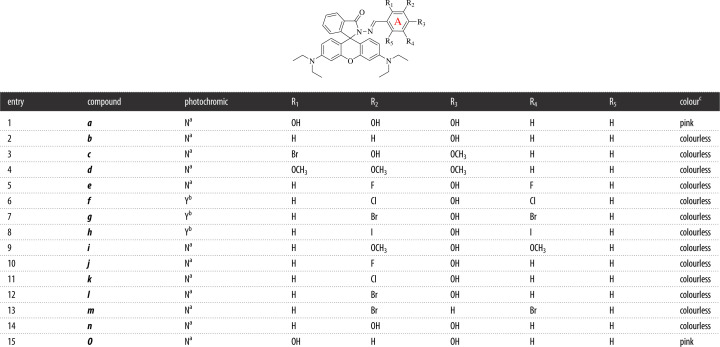

^a^No photochromic property was observed for this compound.

^b^Photochromic compound.

^c^The colour of solution for compounds (20 µM) after treatment with 50 µM Cu^2+^ before light irradiation.

## 2. Experimental section

### 2.1. Synthesis and structural characterization of compounds ***a***–***o***

**Figure 1.**
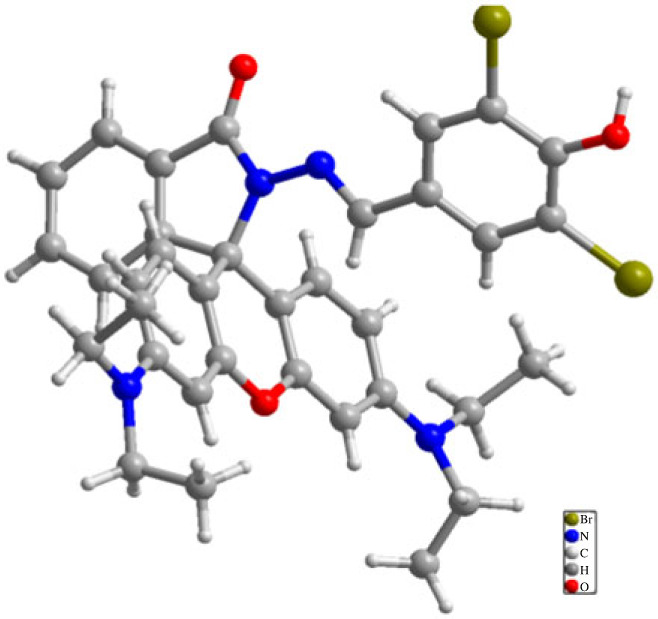
Single crystal structure of compound ***g.***

Chemical regents used in this work were all commercially available and used without further purification (supporting information (ESI), Part I). Compounds ***a***–***o*** were obtained according to the previous reported synthetic methods (ESI, Part II) and fully characterized with instruments such as nuclear magnetic resonance (NMR) (ESI, Part III–IV). Single crystal of compound ***g*** was obtained in ethanol by slowly evaporating the solvent and collected on a Xcalibur Eos Gemini four-circle diffractometer with Mo K*α* radiation (*λ* = 0.71073 Å) at 296 K. The single crystal structure of compound ***g*** is displayed as figure 1 and the single crystal data are listed in the supporting information (ESI, Part II, electronic supplementary material, table S1). The Cambridge Crystallographic Data Centre (CCDC) number of compound ***g*** is 2 142 367. The structures of compounds ***a****–****o*** are displayed in electronic supplementary material, table S2.

### 2.2. Stock solution preparation and photochromic properties study

Stock solutions of compounds ***a****–****o*** were prepared in ethanol with a concentration of 1 mM. Corresponding metal nitrates were used to prepare the metal ions stock solutions in deionized water (Na^+^, K^+^, Zn^2+^, Cd^2+^, Co^2+^, Ni^2+^, Al^3+^, Cu^2+^, Cr^3+^, Fe^3+^, Tb^3+^, Mg^2+^, Ca^2+^, Ba^2+^ and Dy^3+^) with a concentration of 5 mM. Tris–HCl buffer were prepared in ethanol and water (pH = 2–9, 10 mM, *V*_ethanol_ : *V*_water_ = 1 : 1). Compound ***g*** was selected as the model substrate to investigate the Cu^2+^ triggered photochromic properties and applications. All experiments were performed at room temperature unless otherwise specified.

## 3. Results and discussion

### 3.1. Photochromic properties study of compound ***g***

#### 3.1.1. pH effect

**Figure 2.**
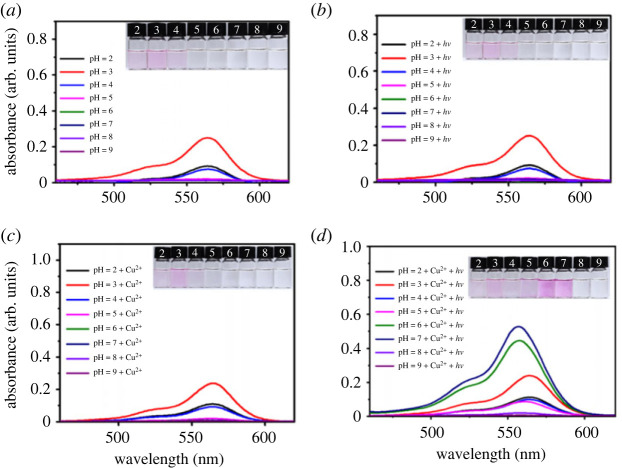
(*a*) UV-vis spectra of ***g*** (10 µM) in Tris-HCl buffer with different pH; (*b*) UV-vis spectra of ***g*** (10 µM) after irradiating with visible light for 10 min in Tris–HCl buffer with different pH; (*c*) UV-vis spectra of ***g*** (10 µM) after treatment with Cu^2+^ (50 µM) in Tris–HCl buffer with different pH; (*d*) UV-vis spectra of ***g*** (10 µM) after treatment with Cu^2+^ (50 µM) and visible light irradiation in Tris–HCl buffer with different pH.

It is well known that the spiral lactam ring in rhodamine derivatives is highly sensitive to the pH changes of the environment. In most cases, the absorbance of rhodamine derivatives at approximately 560 nm would be greatly enhanced accompanied by visible colour change when the spiral lactam ring was opened by external stimulations. Thus, in order to exclude the influence of pH on the photochromic property of compound ***g***, a series of control experiments have been performed (figure 2).

Initially, the UV-vis spectra of 10 µM compound ***g*** in Tris–HCl buffer (pH from 2 to 9) were recorded without external light irradiation. As displayed in figure 2*a*, remarkable absorbance enhancements centred at 560 nm were observed in stronger acidic environments (pH value 2–4). However, no obvious absorption could be recorded in weak acidic, neutral and alkaline environments (pH 5–9). Apparently, the UV-vis test results are consistent with the colour change of the solutions (the insert photographs). Then, the above samples were irradiated with external visible light for 10 min and their UV-vis spectra were recorded in figure 2*b*. Compared with figure 2*a*, no remarkable change could be observed. The above results revealed that the spiral lactam ring in compound ***g*** could be opened by strong acid but not light irradiation in the absence of metal ions.

**Figure 3.**
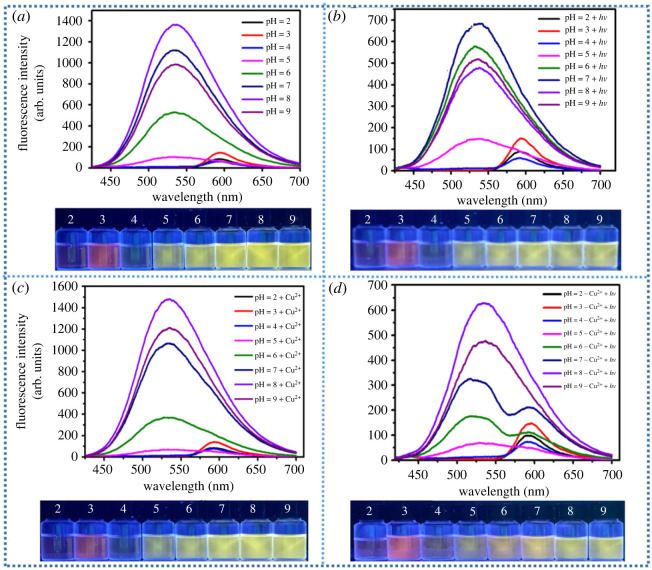
(*a*) Fluorescence spectra and corresponding photograph of ***g*** (10 µM) in Tris–HCl buffer with different pH; (*b*) fluorescence spectra and corresponding photograph of ***g*** (10 µM) after irradiated with visible light for 10 min in Tris–HCl buffer with different pH; (*c*) fluorescence spectra and corresponding photograph of ***g*** (10 µM) after treatment with Cu^2+^ (50 µM) in Tris–HCl buffer with different pH; (*d*) fluorescence spectra and corresponding photograph of ***g*** (10 µM) after treatment with Cu^2+^ (50 µM) and visible light in Tris-HCl buffer with different pH.

The above samples were treated with 50 µM Cu^2+^ in the absence and presence of external light irradiation. As shown in figure 2*c*, the absorbance intensity of samples after treatment with 50 µM Cu^2+^ has no obvious change, suggesting that Cu^2+^ could not trigger the spiral lactam ring opening in the absence of external light stimulation. Interestingly, turn-on colour changes from colourless to pink with absorbance enhancements were observed for samples in buffer with pH 5–7 (figure 2*d*, insert) after irradiating with visible light for 3 min. The maximum enhancement emerged at pH 7.0. However, with further increase of the pH value to 8–9, the photochromic phenomenon would disappear. The above experimental results confirm that Cu^2+^ and pH value both played key roles in the light-induced photochromic of compound ***g*** (figure 2*d*). In addition, fluorescent spectra of the above samples were also recorded to confirm the pH effect (figure 3).

As displayed in photograph 3a (figure 3*a*), compound ***g*** exhibited bright yellow fluorescence emission at pH 5–9 under 365 nm UV-light. While, slight pink fluorescence emissions centred at 580 nm were observed for those samples in stronger acidic buffer (pH 2–4), indicating that the spiral lactam ring of ***g*** was opened in strong acid conditions. No obvious emission wavelength change was observed for the above samples after irradiating with visible light (compared with figure 3*a*), though the fluorescence intensity decreased (figure 3*b*). The above results suggested that only light irradiation could not activate the photochromic process. Next, the fluorescent spectra of the above samples after treatment with 50 µM Cu^2+^ in the absence and presence of light irradiation were recorded and are shown in figure 3*c,d*. Apparently, only addition of Cu^2+^ will not lead to the colour or obvious spectra change of the samples (figure 3*c*), indicating that the interaction between Cu^2+^ and ***g*** is too weak to open the spiral lactam ring. Interestingly, new fluorescence emission peak centred at 580 nm emerged for the above samples at pH 5–7 after irradiation with visible light for 3 min, which is consistent with the spectra and colour changes in figure 2*d*. In addition, in order to exclude the false positive results caused by pH, the following photochromic studies were carried out in Tris-HCl buffer at pH = 7.0.

#### 3.1.2. Trigger selectivity

**Figure 4.**
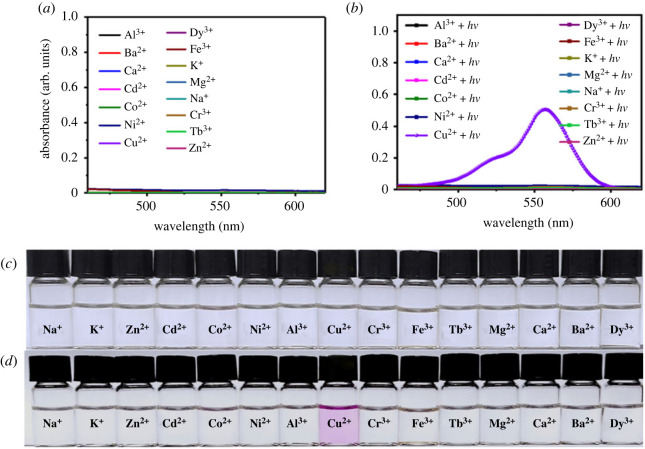
(*a*) Absorbance spectra of ***g*** (10 µM) in Tris–HCl buffer (pH = 7.0) after treatment with 50 µM metal ions in the absence of external light irradiation; (*b*) absorbance spectra of the above samples after irradiated with visible light for 10 min; (*c*) photograph of the above samples before irradiating with visible light; (*d*) photograph of the above samples after irradiating with visible light for 10 min.

It is generally assumed that photochromic systems that could be activated by a special external stimulation are more advantageous than those that could be initiated by multiple external stimulations in applications such as information storage and encryption. In order to reveal the trigger selectivity of this photochromic system, various metal ions including Na^+^, K^+^, Zn^2+^, Cd^2+^, Co^2+^, Ni^2+^, Al^3+^, Cu^2+^, Cr^3+^, Fe^3+^, Tb^3+^, Mg^2+^, Ca^2+^, Ba^2+^ and Dy^3+^ were used to coordinate with ***g*** in Tris-HCl buffer (pH = 7.0). As depicted in figure 4*a*, no remarkable absorbance was observed for ***g*** after treatment with various metal ions without external light irradiation, and the solutions were colourless (figure 4*c*). Interestingly, after irradiation with visible light, only the sample that treatment with Cu^2+^ changed from colourless to pink (figure 4*d*) and the absorbance centred at 560 nm enhanced dramatically (figure 4*b*).

**Figure 5.**
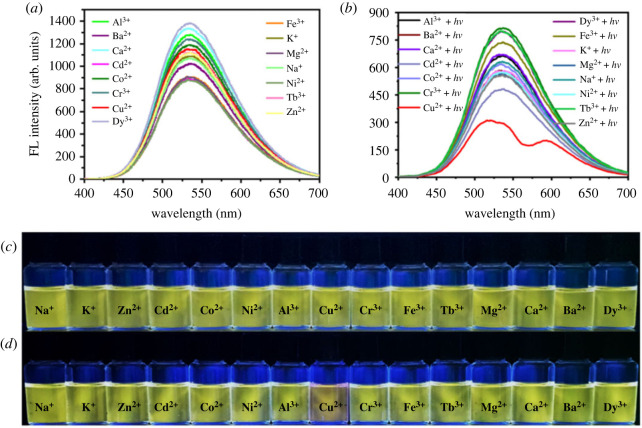
(*a*) Fluorescence spectra of ***g*** (10 µM) in Tris–HCl buffer (pH = 7.0) after treatment with 50 µM metal ions in the absence of external light irradiation; (*b*) fluorescence spectra of ***g*** (10 µM) in Tris–HCl buffer (pH = 7.0) after treatment with 50 µM metal ions and external light irradiation (*λ*_ex_ = 380 nm, slits 5.0 nm/2.5 nm); (*c*) photograph of the above samples before irradiating with visible light under 365 nm UV-light; (*d*) photograph of the above samples after irradiating with visible light for 10 min under 365 nm UV-light.

In order to further confirm the trigger selectivity, fluorescent spectra of ***g*** after treatment with various ions were also recorded. As shown in figure 5*a,b*, among the tested 15 metal ions, only Cu^2+^ could lead to a new fluorescence emission that centred at 580 nm after irradiating with external light, which was consistent with the UV-vis spectra change (figure 4*a,b*). The fluorescence change could also be confirmed by the photograph of the samples under 365 nm UV-light (figure 5*c,d*). In addition, on the basis of the above results, we can conclude that this photochromic system could be specifically triggered by Cu^2+^ in the presence of light irradiation.

#### 3.1.3. Response time study

**Figure 6.**
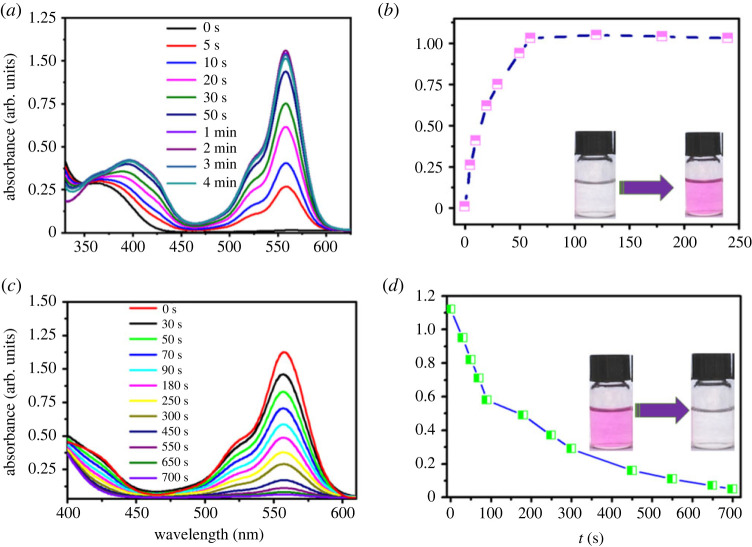
(*a*) Absorbance spectra of ***g*** (10 µM) in Tris–HCl buffer (pH = 7.0) after treatment with 200 µM Cu^2+^ with different irradiation time; (*b*) absorbance intensity centred at 560 nm of the above samples with different irradiation time (inset: the colour change before and after light irradiation); (*c*) absorbance spectra of the above samples after dark thermal bleaching for different time; (*d*) absorbance intensity centred at 560 nm of the above samples with different irradiation time (inset: the colour change before and after thermal bleaching).

An appropriate activation and thermal bleaching rate as well as good fatigue resistance are essential characters for a practical photochromic system. In order to reveal the response rate of this photochromic system to light irradiation and thermal bleaching, the UV-vis spectra of ***g*** after treatment with 200 µM Cu^2+^ were recorded with different irradiation time. As shown in figure 6*a*, there was no obvious absorbance from 450 to 600 nm for the sample in the absence of external light irradiation. Interestingly, a remarkable absorbance enhancement was recorded after 5 s light irradiation for the same sample. Further increasing the irradiation time, the absorbance intensity centred at 560 nm would be enhanced correspondingly. The absorbance reached the maximum intensity after 60 s irradiation and would no longer increase with further extent of the irradiation time (figure 6*b*).

The above tests proved this photochromic system could be activated in 1 min with visible light irradiation, which is beneficial to construct a fast-response photochromic system. To further reveal the thermal bleaching rate of this photochromic system, UV-vis spectra of the fully activated samples after being placed in dark for different time were recorded and are displayed in figure 6*c,d*. As expected, the absorbance intensity decreased gradually with time extended in dark. After approximately 5 min dark treatment, the absorbance intensity centred at 560 nm (figure 6*d*) was approximately equal to the sample that was irradiated with light for 5 s (figure 6*a*). The colour faded to colourless after approximately 700 s, indicating this photochromic system has a moderated thermal bleaching rate, which is an essential character for practical application.

#### 3.1.4. Reversibility and fatigue resistance

**Figure 7.**
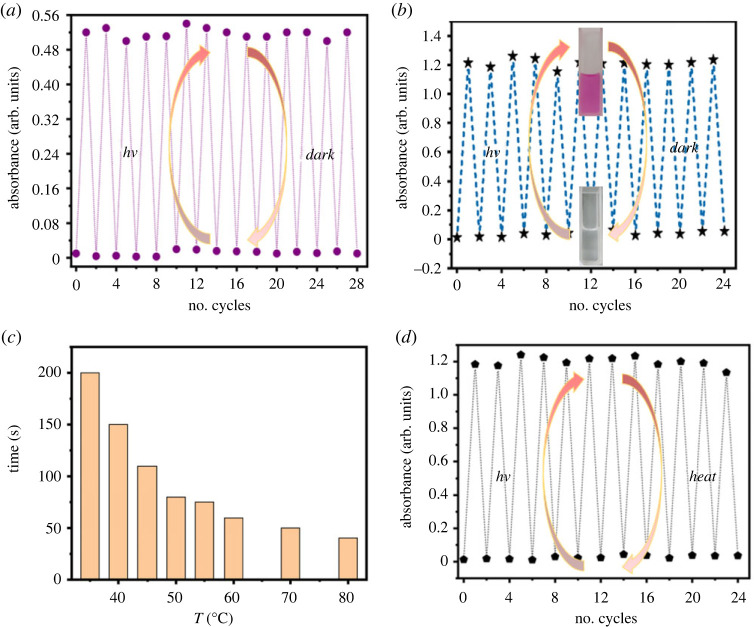
(*a*) Absorbance intensities centred at 560 nm of the photochromic system (10 µM ***g*** and 50 µM Cu^2+^ in 10 mM Tris-HCl buffer, pH = 7.0) after alternately light irradiation for 3 min and dark treatment for 10 min; (*b*) absorbance intensities of the photochromic system centred at 560 nm (20 µM ***g*** and 300 µM Cu^2+^ in 10 mM Tris-HCl buffer, pH = 7.0) after alternately light irradiation for 3 min and dark treatment for 10 min; (*c*) the time needed for dark thermal bleaching under different temperature for this photochromic system (20 µM ***g*** and 200 µM Cu^2+^ in 10 mM Tris–HCl buffer, pH = 7.0); (*d*) rapid thermal bleaching cycles for the photochromic system under 80°C (20 µM ***g*** and 200 µM Cu^2+^ in 10 mM Tris–HCl buffer, pH = 7.0).

Good reversibility and fatigue resistance are the essential criteria for a photochromic system to be used as a reversible photochromic material. Therefore, the absorbance intensity of this photochromic system (10 µM ***g*** and 50 µM Cu^2+^ in 10 mM Tris–HCl buffer, pH = 7.0) centred at 560 nm was recorded with alternately light irradiation for 3 min and dark treatment for 10 min. As shown in figure 7*a*, the absorbance intensities reversed between 0.01 and 0.52 for 14 times without any apparent degradation. In order to reveal if the reversibility would be influenced by the existence of excess Cu^2+^, the absorbance intensity of this photochromic system (20 µM ***g*** and 300 µM Cu^2+^ in 10 mM Tris-HCl buffer, pH = 7.0) centred at 560 nm was recorded according to the above method. As displayed in figure 7*b*, no obvious change was observed for the maximum absorbance intensity (after light irradiation) and the minimum absorbance intensity (after dark thermal bleaching) of this photochromic system after 12 cycle tests.

The dark thermal bleaching of this photochromic system (20 µM ***g*** and 200 µM Cu^2+^ in 10 mM Tris-HCl buffer, pH = 7.0) would take approximately 10 min at room temperature; we suspect that the bleaching rate would be accelerated with external heating. Therefore, the time needed for thermal bleaching under different temperature for this photochromic system (20 µM ***g*** and 200 µM Cu^2+^ in 10 mM Tris-HCl buffer, pH = 7.0) was recorded. As shown in figure 7*c*, the thermal bleaching rate greatly accelerated with the increase of temperature, and the bleaching time needed at 80°C was approximately 40 s. In addition, the fatigue resistance with room temperature light activation and thermal bleaching at 80°C was also revealed. As shown in figure 7*d*, the absorbance intensities kept constant after 12 cycles light activation and thermal bleaching, indicating the good fatigue resistance of this photochromic system (20 µM ***g*** and 200 µM Cu^2+^ in 10 mM Tris-HCl buffer, pH = 7.0).

### 3.2. Substituent modulation effect study

**Figure 8.**
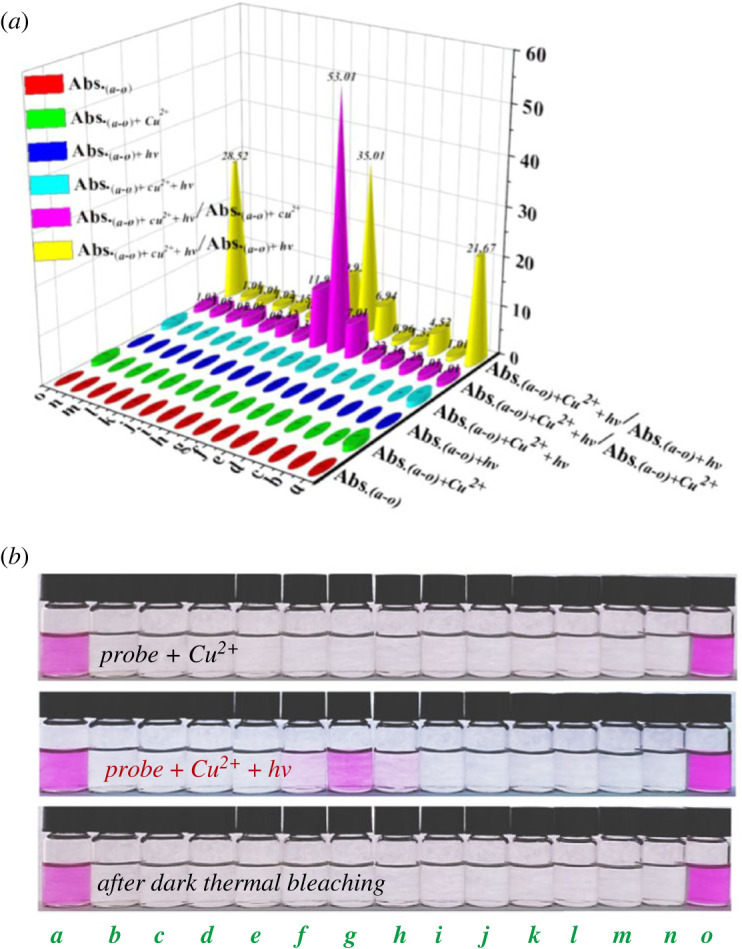
(*a*) Absorbance intensity (or intensity ratio) of 20 µM compounds ***a***–***o*** with 50 µM Cu^2+^ (10 mM Tris-HCl buffer, pH = 7.0) under different conditions; (*b*) photographs of compounds ***a***–***o*** in the presence of Cu^2+^ under different conditions (10 mM Tris–HCl buffer, pH = 7.0).

To the best of our knowledge, the modulation effect of substituents on photochromic property of RhBHH derivatives has not been systematically revealed yet. Therefore, 15 RhBHH derivatives (table 1, compounds ***a***–***o***, structures in electronic supplementary material, table S2) with various substituents at different position of ring **A** have been synthesized and their photochromic property triggered by Cu^2+^ was systematically studied by using molecular engineering strategy (table 1). The UV-vis spectra of compounds ***a****–****o*** under different conditions were recorded respectively (electronic supplementary material, figures S17–S32, UV-vis spectra of compounds ***a***–***o***, UV-vis spectra of compounds ***a***–***o*** + ***hv***, UV-vis spectra of compounds ***a***–***o*** + Cu^2+^, UV-vis spectra of compounds ***a***–***o*** + Cu^2+^ + ***hv***). The corresponding absorbance intensity (or intensity ratio) centred at 560 nm is summarized in figure 8*a* and the corresponding photographs are shown in figure 8*b*.

Interestingly, irreversible colour change was recorded for compounds that have a hydroxyl group at R_1_ position in the absence or presence of external light irradiation (compound ***a*** and ***o***, table 1, entries 1 and 15, and figure 8*b*), while no observable colour change could be recorded for those compounds with a halogen or methoxyl group at R_1_ position whether the light irradiation existed or not (table 1, entries 3 and 4, and figure 8*b*). To our surprise, reversible photochromic phenomenon triggered by Cu^2+^ was observed for compounds ***f***–***h*** which have hydroxyl group at R_3_ position and halogen groups at R_2_ and R_4_ position (table 1, entries 6–8, R_3_ = OH, R_2_ = R_4_ = Cl, Br, I). While, no photochromic phenomenon could be observed for compound ***m*** (table 1, entry 13, R_2_ = R_4_ = Br, R_3_ = H). Apparently, the existence of a *para*-hydroxyl group (R_3_ = OH) was extremely important for this Cu^2+^-triggered photochromic system. Besides, the nature of R_2_ and R_4_ also has great influence on the photochromic property. For example, although the existence of hydroxyl group at R_3_ position for compound ***b*** (table 1, entry 2)*, **e*** (table 1, entry 5), ***i***–***l*** (table 1, entries 9–12)*,* and ***n*** (table 1, entry 14), no photochromic phenomenon could be observed. It is noteworthy that the kind of halogen atom at R_2_ and R_4_ positions exhibited important modulation effect. For instance, compounds ***g*** (table 1, entry 7, R_3_ = OH, R_2_ = R_4_ = Br, figure 8*b*) showed typical reversible photochromic property; however, no photochromic property was observed for compound ***e*** (table 1, entry 5, R_3_ = OH, R_2_ = R_4_ = F, figure 8*b*). On the other hand, the highly specific response could be reflected by the absorbance intensity ratio of different compounds (Abs._(*a−o*)*+Cu*_*^2+^_+hv_/*Abs._(*a−o*)*+Cu*_*^2+^* and Abs*.*_(*a−o*)*+Cu*_^2+^*_+hv_**/***Abs*.*_(*a−o*)*+hv*_). As depicted in figure 8*a*, the intensity ratio of compounds ***f***–***h*** (Abs._(*a−o*)*+Cu*_*^2+^_+hv_/*Abs._(*a−o*)*+Cu*_*^2+^*) is significantly higher than other compounds (the intensity ratio is 53.01 for compound ***g***), indicating the system constructed by compounds ***f***–***h*** and Cu^2+^ is highly sensitive to the light irradiation. The intensity ratio (Abs*.*_(*a−o*)*+Cu*_*^2+^_+hv_**/***Abs*.*_(*a−o*)*+hv*_) of ***a***, ***f***–***h*** and ***o*** is significantly higher than other compounds, indicating compounds ***a***, ***f***–***h*** and ***o*** have non-negligible interaction with Cu^2+^.

However, the absorbance intensity ratio (Abs._(*a−o*)*+Cu*_*^2+^_+hv_/*Abs._(*a−o*)*+Cu*_*^2+^*) of compounds ***a*** and ***o*** is near to 1.0, suggesting the existence of strong interaction between compounds ***a***, ***o*** and Cu^2+^, which will lead to the insensitive response to external light irradiation and irreversible colour change. Thus, the photochromic property of RhBHH derivatives could be modulated by changing the substituent groups of ring **A** (table 1).

### 3.3. Structure photochromic response relationship study and plausible mechanism

**Figure 9.**
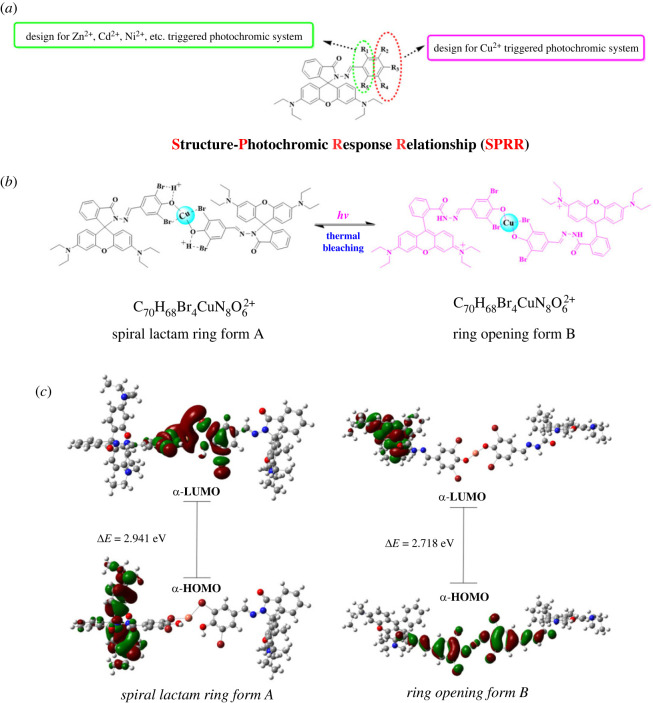
(*a*) A summary of the structure–response relationship (SRR) for RhBHH photochromic systems; (*b*) a plausible mechanism for the Cu^2+^ triggered photochromic phenomenon of compound ***g***; (c) DFT-optimized structure and molecular orbital plots of the HOMO and LUMO of spiral lactam ring form A and spiral lactam ring opening form B.

With the above results in hand and referring to the previous works [18–26], the SPRR of RhBHH photochromic systems could be obtained. First of all, a hydroxyl group is necessary for most of the RhBHH-based photochromic system. Secondly, an *ortho*-hydroxyl group is vitally important for the Ni^2+^, Zn^2+^, Cd^2+^ etc.-triggered photochromic system, but not suitable for Cu^2+^-triggered photochromic system (figure 9*a*, R_1_ or R_5_) due to the strong chelate effect of Cu^2+^ [33–35]. Moreover, a *para*-hydroxyl group and two adjacent halogen atoms (figure 9*a*, R_2_ = R = Cl, Br, I) are the key structure for Cu^2+^-triggered photochromic system. No photochromic was observed for compound ***e*** (table 1, entry 5, R_3_ = OH, R_2_ = R_4_ = F, figure 8*b*), which might be ascribed to the strong electronegativity of fluorine atom leading to the poor coordination ability and hampering the formation of light-sensitive complex (figure 9*b*, complex A). In addition, a combination of Cu^2+^ and RhBHH derivatives that have an *ortho*-hydroxyl group (figure 9*a*, R_1_ or R_5_) will lead to instant and irreversible colour change (colourless to pink or red), which is independent of light irradiation.

According to the above results and analysis, a plausible mechanism has been proposed in figure 9*b*. The coordination between the *para*-hydroxyl group in compound ***g*** and Cu^2+^ leads to the formation of a colourless complex A (the spiral lactam ring form A, figure 9*b*) in the absence of external light irradiation. One of the two adjacent halogen atoms (figure 9*b*, R_2_ and R_4_) coordinate to Cu^2+^ with a weak Cu-Br bond to guarantee the stability of the complex A (the spiral lactam ring form A, figure 9*b*). Intra-molecular hydrogen bonds might be formed between another halogen atom (figure 9*b*, R_2_ or R_4_) and hydroxyl hydrogen to further stabilize complex A according to the Fourier transform infrared (FT-IR) tests and DFT calculation (electronic supplementary material, figure S33, a broad peak from 3700 to 3200 cm^−1^ was recorded before light irradiation, while a sharp peak centred at 3360 cm^−1^ appeared after light irradiation; electronic supplementary material, figure S35, the bond distance of H-Br is 2.48 Å in complex A). The spiral lactam ring opened with external light stimulation leading to the formation of the spiral ring opening complex B (the sharp peak centred at 3360 cm^−1^ after light irradiation could be ascribed to the vibration of N-H). Remarkable colour change from colourless to pink was observed along with the ring opening process. In addition, the formation of complex (A or B) could be confirmed with the ESI-MS test. As shown in electronic supplementary material, figure S34, a peak 749.9 could be observed in the ESI-MS spectra which could be ascribed to the complex (complex A or B, C_70_H_68_Br_4_CuN_8_O_6_^2+^, *m/z*: calcd. 749.6; found 749.9).

The plausible mechanism of this revisable Cu^2+^ triggered photochromic and thermal bleaching phenomenon was further studied by using theoretical calculations method (DFT calculation) [36]. Optimized geometries of the spiral lactam ring form A (figure 9*b*) and spiral lactam ring opening form B (figure 9*b*) are displayed in electronic supplementary material, figure S35. As the calculated results indicate, when the photochromic system changed from A to B, there will be a 47.54 kcal mol^−1^ energy release (electronic supplementary material, figure S35, ΔH = −47.54 kcal mol^−1^, ΔG = −50.47 kcal mol^−1^). This explains why the spiral lactam ring form A could be activated in a very short time (approx. 5 s) by visible light which has less energy, while the thermal bleaching needs more than 10 min or heating at higher temperature. Moreover, the bond distances of Cu-O, Cu-Br, H-Br are 2.15, 2.51 and 2.48 Å, respectively, in the spiral lactam ring form A (electronic supplementary material, figure S35). However, the bond distances of Cu-O and Cu-Br remarkably changed to 1.82 and 3.08 Å, respectively, in the spiral lactam ring opening form B (electronic supplementary material, figure S35). The above results suggesting that Cu-O bond is strengthened in the light induced colour change process, while the Cu-Br bond is weakened. We can deduce from the above results that the halogen groups play an import role in the photochromic modulation process.

In addition, the molecular orbital plots of the HOMO and LUMO of form A and form B are displayed in figure 9*c*. Interestingly, great changes could be observed by comparison of the molecular orbital distribution, for example, the HOMO and LUMO spread in different regions of the structure both in form A and form B. In the spiral lactam ring form A, the HOMO is spread mainly on the xanthene core structure, while the LUMO spread over the Cu^2+^ coordination area. In the ring opening structure (form B), the HOMO mainly situates on the Cu^2+^ coordination area of complex B, while the LUMO locates mainly on the xanthene core structure. The above molecular orbital change is in consonance with the electron distribution that is displayed in figure 9*b*. Moreover, compared with form A, the ring opening structure (form B) has a smaller HOMO–LUMO gap, which would result in the red-shift of the absorption spectra (electronic supplementary material, figure S36). In addition, the theoretical calculations results are in consonance with FT-IR analysis and ESI-MS study (electronic supplementary material, figures S33–S34).

### 3.4. Potential applications

#### 3.4.1. Photochromic glass

**Figure 10.**
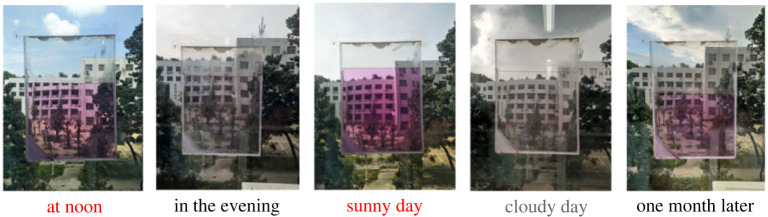
Photographs of the photochromic glass prepared with the developed photochromic system (20 µM ***g*** and 50 µM Cu^2+^ in 10 mM Tris–HCl buffer, pH = 7.0) under different conditions (the photograph for the same photochromic glass that was used for one month was obtained in a sunny day at noon).

The photochromic system constructed with compound ***g*** and Cu^2+^ has been proved highly sensitive to the visible light stimulation. Inspired by the above results, photochromic glass was prepared and used in our laboratory. As shown in figure 10, the photochromic glass exhibited pink colour when the external light was strong (at noon), while the pink colour faded automatically with the coming of evening (weak external light stimulation). Interestingly, this photochromic glass could work smartly. For instance, the colour of this glass would be changed with the weather variation. As displayed in the photograph, the colour of the as-prepared glass reversibly changed from pink to colourless when the weather changed between sunny day and cloudy day. In addition, this photochromic glass displayed good fatigue resistance. As shown in the last photograph, the photochromic glass still works smartly and no obvious degradation could be observed after one month tests (figure 10).

#### 3.4.2. Special security ink with light readout and dark/heat bleaching

**Figure 11.**
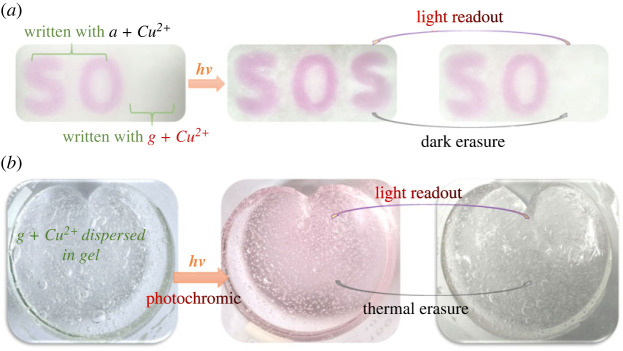
Photographs of applications as special security ink in the matrix of silica and gel.

With the substituent group modulation study results in hand, the initial attempts to use compounds ***a*** and ***g*** as special security inks in the matrix of silica were performed. As shown in figure 11, the first two letters of the word ‘SOS’ was written with the mixture of compounds ***a*** and Cu^2+^, while the last letter ‘S’ was written with the mixture of compounds ***g*** and Cu^2+^. Interestingly, two different words with totally different meaning (SOS and SO) were observed alternatively with light irradiation and dark thermal bleaching. In addition, photochromic gel with light readout and thermal erasure property could be prepared by dispersing this photochromic system (compounds ***g*** and Cu^2+^) into the gel (figure 11*b*). Thus, the developed photochromic systems might be a promising candidate material for information storage and encryption with Cu^2+^ as the key.

#### 3.4.3. Molecular logical gate

**Figure 12.**
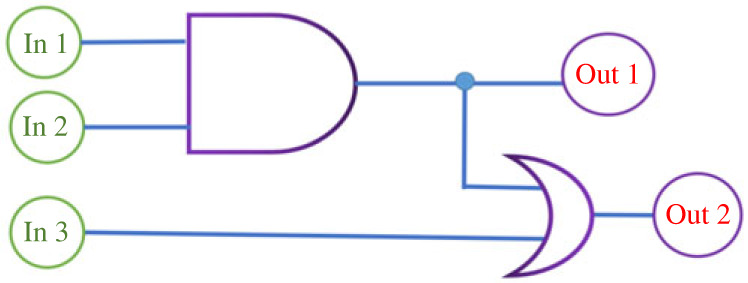
Molecular logic gate based on compounds ***a*** and ***g*** by using the colour change (colourless to pink or red) as output (10 mM Tris-HCl buffer, pH = 7.0).

Inspired by the remarkable difference in response of ***a*** and ***g*** after treatment with Cu^2+^ in the absence and presence of external light irradiation, a molecular logical gate with three input signals and two output signals is constructed based on compounds ***a*** and ***g***. Input 1 is defined as the presence (In 1 = 1) or absence (In 1 = 0) of Cu^2+^ (table 2). Input 2 is defined as the presence of compound ***g*** (In 2 = 1) or compound ***a*** (In 2 = 0) (table 2). Input 3 is defined as the presence (In 3 = 1) or absence (In 3 = 0) of external light irradiation (table 2). The visible colour change from colourless to pink is defined as ‘1’ for the output signals (Out 1, Out 2), while no obvious colour change is defined as ‘0’ (table 2). As depicted in table 2 and figure 12, pink or red colour output 1 and output 2 would be observed at the same time when input In 1 = 1 and In 2 = 0 (compound ***a*** and Cu^2+^), regardless of input 3 (In 3 = 0 or 1). Interestingly, when output 1 and output 2 give the positive signal (Out 1 = Out 2 = 1) at the same time, it means the tested compound is not photochromic (In 1 = 1, In 2 = 0, In 3 = 0 or 1). When output 2 give the positive signal and output 1 give the negative signal (Out 1 = 0, Out 2 = 1), it means the tested compound is photochromic (In 1 = 1, In 2 = 1, In 3 = 1).

**Table 2.** Truth table of the logic gates based on Cu^2+^, compounds ***a****, **g*** and visible light irradiation. In 1 = 1 when in the presence of Cu^2+^; = 0 when in the absence of Cu^2+^. In 2 = 1 when compound ***g*** was used; = 0 when compound ***a*** was used. In 3 = 1 when irradiate with visible light; = 0 when no light irradiation.

input			output	
In 1 (Cu^2+^)	In 2 (compounds)	In 3 (*hv*)	Out 1	Out 2
0	0	0	0	0
0	1	0	0	0
0	0	1	0	0
0	1	1	0	0
1	0	0	1	1
1	1	0	0	0
1	0	1	1	1
1	1	1	0	1

#### 3.4.4. Two-dimensional code security information storage device

**Figure 13.**
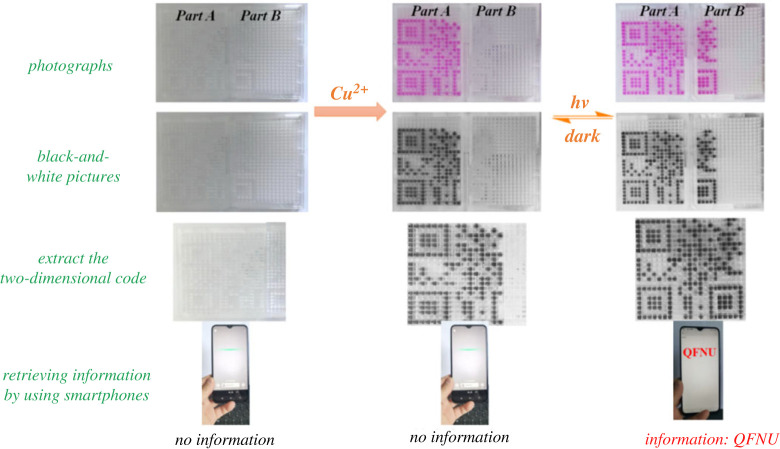
Two-dimensional code security information storage device based on the photochromic system.

The potential application of the above photochromic system in the security information storage, encryption and authentication was further revealed with a two-dimensional code. The two-dimensional code was encoded with the information QFNU and fabricated with compounds ***a*** (part A) and ***g*** (part B) (figure 13). Interestingly, the encoded information could be retrieved by using smartphone with applications such as Wechat under specific condition. For instance, no information could be extracted in the absence of Cu^2+^. Although, an incomplete two-dimensional code could be obtained after addition of Cu^2+^, no valid information could be extracted. While, a complete two-dimensional code could be obtained after light irradiation, and the encoded information QFNU could be successfully extracted. In addition, the encoded information QFNU could be facilely hidden after dark bleaching. Thus, the as-prepared two-dimensional code system exhibited good potential for security information storage and encryption by using Cu^2+^ as the key.

## 4. Conclusion

In summary, a novel Cu^2+^-triggered photochromic system and its applications were revealed. Fifteen RhBHH derivatives with different substituents were synthesized and three of them exhibited Cu^2+^-triggered photochromic property. The substituent group modulation effect and structure photochromic response relationship (SPRR) of RhBHH-based photochromic system have been systematically studied by using molecular engineering strategy. Different from the most reported photochromic system, the developed photochromic system could be highly selective and sensitive triggered by Cu^2+^ in the presence of visible light irradiation. Good reversible photochromic phenomenon was observed for this photochromic system after stimulating with visible light irradiation and dark (or heat) bleaching, consecutively. A plausible mechanism was proposed based on the substituent group modulation study, DFT calculations and spectra tests such as FT-IR and ESI-MS. Potential application studies confirmed that this photochromic system might be a promising candidate for the preparation of photochromic glass, special security ink, molecular logic gate and security information storage device. In addition, the newly discovered substituent modulating effect might be used as a guiding principle for the design of RhBHH-based photochromic materials in the future.

## Data Availability

The data are provided in electronic supplementary material [37].
